# Supplementary figures and images for: DC/L-SIGN recognition of spike glycoprotein promotes SARS-CoV-2 trans-infection and can be inhibited by a glycomimetic antagonist
Source: PLoS Pathog. 2021 May 20;17(5):e1009576. doi: 10.1371/journal.ppat.1009576 (PMC8136665; doi:10.1371/journal.ppat.1009576)

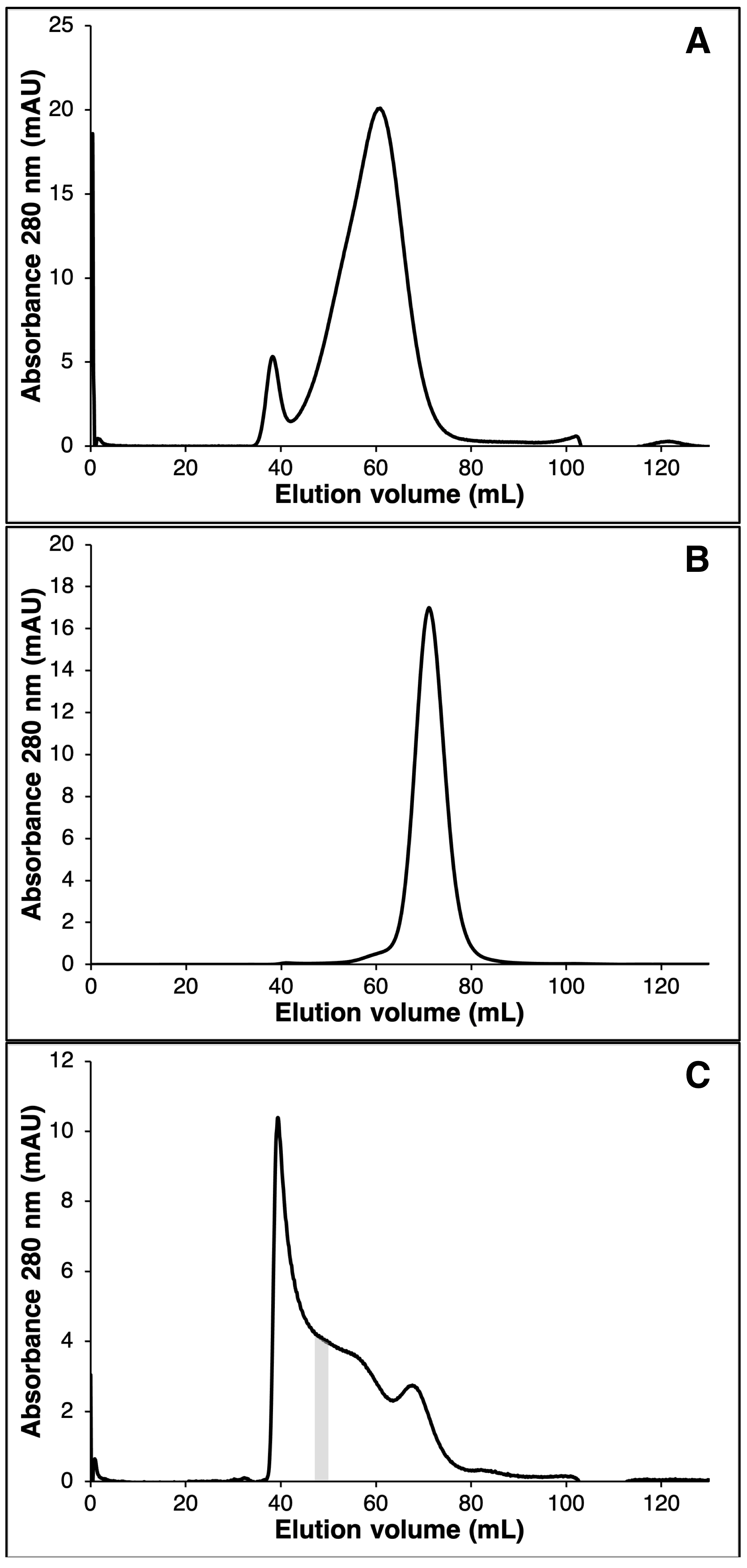

Supplement: S1 Fig — Chromatograms of Superose 6 elution of SARS-CoV-2 Spike (A), DC-SIGN ECD (B) and mix of DC-SIGN ECD and SARS-CoV-2 Spike (C). In panel C, peak at 68 mL correspond to DC-SIGN ECD, which is in excess, and shouldered peak from 40 to 60 mL correspond to spike protein interacting with various numbers of DC-SIGN ECD. Fractions used for Electron Microscopy micrographs of DC-SIGN/SARS-CoV-2 Spike protein complexes (Fig 3C) are represented by the gray square (48 to 50 mL). (TIF) [file ppat.1009576.s001.tif]

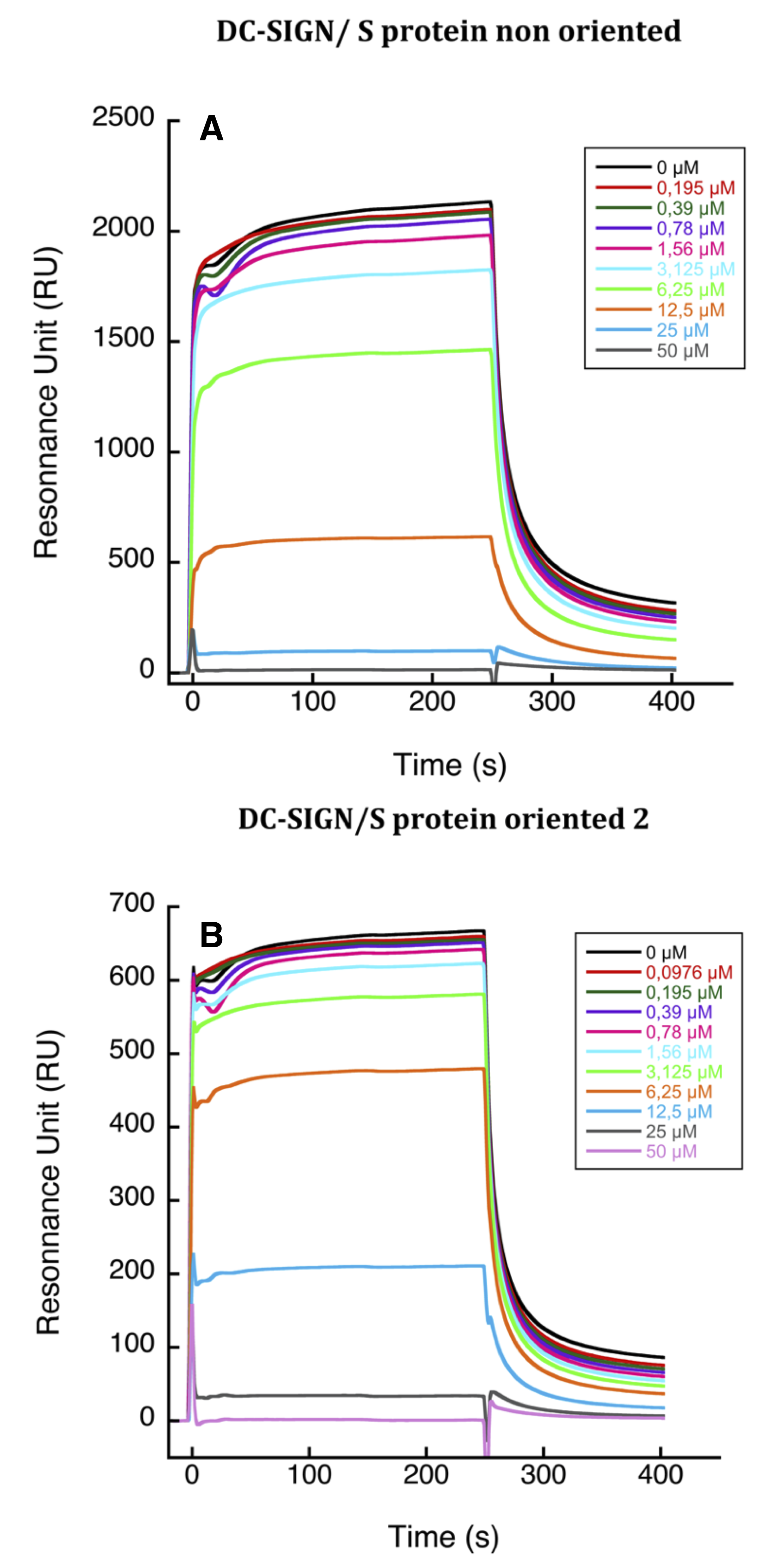

Supplement: S2 Fig — In panel C, peak at 68 mL correspond to DC-SIGN ECD, which is in excess, and shouldered peak from 40 to 60 mL correspond to spike protein interacting with various numbers of DC-SIGN ECD. Fractions used for Electron Microscopy micrographs of DC-SIGN/SARS-CoV-2 Spike protein complexes (Fig 3C) are represented by the gray square (48 to 50 mL). (TIF) [file ppat.1009576.s002.tif]

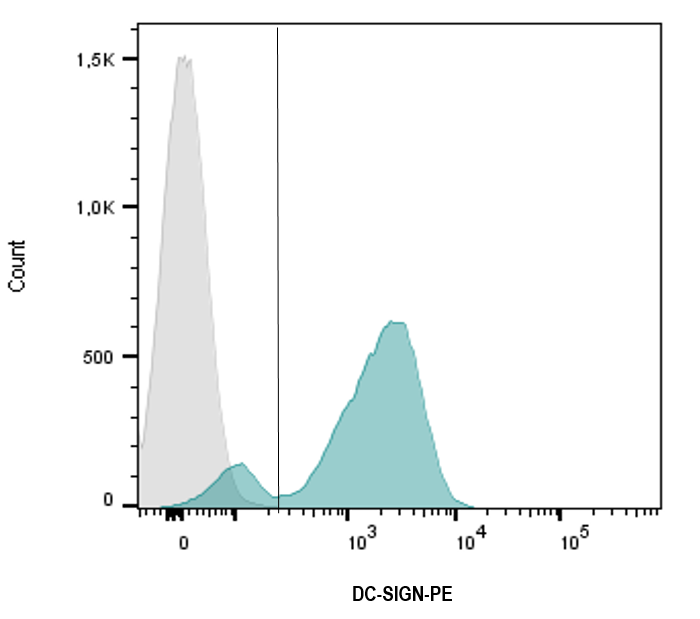

Supplement: S3 Fig — The colored cell population represents MDDCs stained with antibody Anti-DC-SIGN_PE. In grey unstained MDDCs. DC-SIGN expression was analyzed by flow cytometry in a BD FACS Canto II Cell Analyzer with FlowJo V10.7.1 software. (TIF) [file ppat.1009576.s003.tif]
