# Supplementary material for: DC/L-SIGN recognition of spike glycoprotein promotes SARS-CoV-2 trans-infection and can be inhibited by a glycomimetic antagonist
Source: PLoS Pathog. 2021 May 20;17(5):e1009576. doi: 10.1371/journal.ppat.1009576 (PMC8136665; doi:10.1371/journal.ppat.1009576)
Supplement: Attachment — Submitted filename: Point by point answers to reviewers-final.pdf [file ppat.1009576.s004.pdf]

Point by point answers to reviewers

Reviewer #1: This is a strong study which mixes multiple experimental approaches to suggest that DC-SIGN and L-SIGN are important attachment receptors which mediate SARS-CoV-2 trans infection. EM, surface plasmon resonance, bioinformatics, and cell culture models all complement each other nicely.

I would have liked some data if available showing the location of the virus prior to trans infection. As mentioned in the manuscript there is evidence that the virus is contained in a non-endolysosomal compartment in the dendritic cell prior to trans-infection. Did the authors consider EM experiments to demonstrate this?

In this work we mainly focused on the characterization of the capacity of CLRs to recognize and interact with SARS-CoV-2 Spike protein and on their potential role in the spreading of the infection, which led us to highlighting the trans-infection process.
We appreciate the suggestion and share your curiosity about the details of this process and the location of the virus before trans infection.
Is it virus concentration and storage at cell surface, or is this a process involving internalization of virus into a specific compartment before re-exposure for trans infection? Such questions have been already examined in the context of the HIV virus transmission and proved to be complex to discriminate (Geijtenbeek (2000) Cell, Kwon (2002) Immunity). We agree that this is the next step in this work and we will make efforts to use EM to shed light on the problem, but we are not in the position to do this within the timing of the revision. This is mainly because the type of experiments suggested by the referee (characterization by immunolocalization and using EM) are not a routine type of experiment within our institute. We will need to develop this more specifically for this project in the weeks and months to come and even maybe to incorporate new collaborators.

Reviewer #2: Thépaut M et al., propose that DC/L-SIGN, two C-type lectin receptors (CLRs), present in immune cells such as macrophages and dendritic cells in the mucosa and respiratory tissue, serve as attachment points for SARS-CoV-2. These CLRs could recognize glycan sites on Spike. While these immune cells are not infected, they can direct the attached viruses to the surrounding cells that are then infected by a process called trans-infection. The authors showed by surface plasmon resonance (SPR) that when put together, purified DC-SIGN and L-SIGN can associate with Spike from SARS-CoV-2. Electron Microscopy experiments showed the complexes formed by DC-SIGN and Spike and by LC-SIGN and Spike, which are, most of the time, at a stoichiometric ratio of 1:1. Monocyte-derived dendritic cells (MDDCs), and M2 monocyte derived macrophages (M2-MDM), which are cells known to express DC/L-SIGN, were used for the trans-infection experiments, as well as modified Jurkat cells expressing or not DC/L-SIGN. These cells were incubated with VSV/SARS-CoV-2 pseudotyped viruses, that contains firefly luciferase, and then co-cultured with Vero E6 cells. Trans-infection in Vero E6 cells was monitored by the measure of luciferase activity. Authors showed that upon co-culture, pseudoviruses were able to infect Vero E6 cells and this was inhibited using an anti-DC/L-SIGN antibody, suggesting that cells expressing these CLRs can indeed bind the virus and carry it to other cells that can be infected. Moreover, interaction of DC-SIGN with Spike was also inhibited with Polyman26 (PM26), a multivalent glycomimetic mannoside and antagonist of DC-SIGN, which confirms that DC-SIGN recognizes glycans on Spike, as suggested by the authors. Finally, PM26 was also able to inhibit trans-infection on Vero E6 cells.

The subject of this research is of high interest because of the actual pandemic and the potential use of inhibitors of a potential trans-infection phenomenon occurring in the lungs. However, the trans-infection experiments should have been done with a more physiological cell model. Non-human Vero E6 cells are not advised to evaluate SARS-CoV-2 infection of the host. Respiratory cell types of human origin would be an appropriate model. Also, while experiments with pseudotyped viruses have allowed us to learn a lot about SARS-CoV-2 infection, isolated viruses would have also been a better model, in particular for experiments based on the interaction of the virion with the cell.

As asked by the reviewer, new direct infection and trans-infection experiments have been conducted using now authentic SARS-CoV2 and using Calu3, which is a human lung cell line. These experiments confirmed the results previously obtained using pseudo-typed virus. See Figure 7 in the new version of the article.

Reviewer #3: In this study, Thépaut and colleagues report a potential contribution of C-type lectin receptors in SARS-CoV-2 trans-infection of ACE2-expressing cells. For this, they expressed and purified a soluble SARS-CoV-2 Spike (S) stabilized ectodomain protein and performed SPR experiments using soluble multimeric C-type lectin receptors (DC-SIGN, L-SIGN, MGL, and langerin). They found that they all bind with the S ectodomain to different extent and that, although not functioning as entry/triggering receptors, the C-type lectins can mediate trans-infection of VSV pseudotypes from antigen presenting cells to susceptible Vero E6 cells. Although the study is interesting and reveal a potentially important mechanism of infection, the study falls short in characterizing the interaction of SARS-CoV-2 S with C-type lectin receptors and comparing to other viral glycoproteins, and the mechanism by which trans-infection occurs.

Part II – Major Issues: Key Experiments Required for Acceptance

Please use this section to detail the key new experiments or modifications of existing experiments that should be absolutely required to validate study conclusions.

Generally, there should be no more than 3 such required experiments or major modifications for a "Major Revision" recommendation. If more than 3 experiments are necessary to validate the study conclusions, then you are encouraged to recommend "Reject".

Reviewer #1: No further experiments are absolutely needed. Please see the above. I think the data as shown demonstrate that like SARS, trans-infection, mediated by DCL-SIGN, is something that does occur, at least in vitro.

Reviewer #2: 1- First, all experiments are performed with pseudoviruses as a surrogate of virus infection. The authors are interested in DC/L-SIGN as potential attachment points for SARS-CoV-2 (not as an entry receptor). It is known that the virus envelope or other proteins present on the surface may impact the interaction of the virus with host cells. N-glycan sites are not restricted to Spike and could play a role in interaction with DC/L-SIGN proteins. Use of Spike mutant impairing glycosylation are required to strengthen the conclusion that DC/L-SIGN interacts with glycans on Spike. Additionally, it is difficult to know at which extend the results presented in this study are relevant to an infection with the whole virus. If possible, some of the experiments should be done with isolated infectious SARS-CoV-2 virus?

As answered above, new experiments have been performed using authentic SARS-CoV-2 whole virus and confirming the data presented with the pseudotyped virus assay.

2- The second major comment is related to the use of Vero E6 cells for the trans-infection experiments. Vero E6 are not an appropriate model for this particular type of study. Particularly, the entire paper, the authors refer to dissemination of the infection in the respiratory tissue. If the authors want to claim demonstration of dissemination of the infection in respiratory tissue, key experiments should be done at a minimum using a respiratory cell line (Calu-3, A549 expressing ACE2, or other respiratory cell lines expressing ACE2) or event better primary cells.
If not possible, the conclusions and phrasing of the interpretation need to be revised. The scope of the current results is less significant than claimed by the authors.

As asked by the reviewer, experiment using Calu3 cells have been done and in addition using the authentic virus. Same results have been observed, confirming previous data.

3- The authors state that DC/L-SIGN “can also internalize viral particles into cells for storage in non-lysosomal compartments and subsequent transfer to susceptible cells in the process recognized as trans-infection”. The authors claim that it is the main process involved in the trans-infection observed in their study between immune cells and Vero E6 cells. However, the authors do not show evidence of viral particles internalization. Experiment showing that this is really happening are required to support this claim.

This sentence was not a claim regarding to the mechanism of transmission for SARS-CoV-2 identified in this work but a reference to other studies on other virus (Ebola and HIV, corresponding to the two associated references). We used this statement just to introduce the fact that DC/L-SIGN were previously identified as factors in several types of viral transmission processes. To avoid this misunderstanding, we replaced the original sentence (page 17):

> *“DC/L-SIGN are known to enhance viral uptake for direct infection in the process referred to as cis-infection and can also internalize viral particles into cells for storage in non-lysosomal compartments and subsequent transfer to susceptible cells in the process recognized as trans-infection”.*

By the following one, that is more clearly an introductive sentence and do not emphasize on the internalization hypothesis.

> *“With other viruses, DC/L-SIGN are known to enhance viral uptake for direct infection in the process referred to as cis-infection or to allow subsequent transfer to susceptible cells, in the process recognized as trans-infection”*

Reviewer #3: One major shortcoming of this study is the <u>lack of validation with native virus</u>. Indeed, the glycosylation of S onto native virions might be different than the recombinant secreted ectodomain and even from VSV pseudotypes. For instance, the formation of the membrane-associated viral factories and budding at the ERGIC followed by exocytosis might have an effect on glycosylation that could translate in differential binding to C-type lectin receptors.

Validation with native virus added (see above)

In addition, the SPR data are difficult to reconcile, especially given the difference in binding for DC-SIGN with the non-oriented and oriented Spike ectodomain.

We do not understand the comment of the referee on this point, since we **do not** see any significant difference of DC-SIGN binding in the two situations (see table 1).

Observing a major difference between non-oriented and oriented Spike ectodomain would have suggested involvement of a specific protein epitope, that could be masked in one of the functionalization modes. Here, both situations give similar K_d values, which is consistent with multiple possible sites of binding, in coherence with glycosylation sites spread all over the spike surface and thus accessible whatever the orientation. This observation is a first strong argument to say that the lectin receptors recognize glycans on the surface, which is their function.

The Kd values are relatively high

An explanation about the high Kd: individual DC-SIGN CRDs have affinity in the mM range for the target oligosaccharides. Here, we used oligomeric ectodomains of each C-Type Lectin - going from trimer (langerin and MGL) to tetramer (DC-SIGN and L-SIGN)- and thus generating avidity properties thanks to multiple attachment. However, the CLRs are not immobilized, but injected in the flow cell and in this set up the avidity is just limited to the number of CRDs presented by individual oligomeric Lectin. A much higher avidity, and thus a lower Kd_{app} , would have been generated by immobilizing the CLRs. This would have generated surface avidity involving much more CRDs from numerous adjacent CLRs. It was not the set up chosen here for technical limitations and also to allow screening of different lectin receptors thanks to the use of a Spike surfaces. In the end, the affinity obtained here, with soluble oligomeric ectodomain of C-type lectin, is totally consistent with previous observations with other immobilized glycoconjugates, such as BSA-Man. With 3 to 4 CRDs per lectin, it is always an avidity in the μM range, i.e. 3 orders of magnitude more than individual CRDs (mM), but less than the avidity that could have been obtained by a surface presentation of lectins.

and the lack of saturation for Langerin is problematic.

We do not think that the lack of saturation for Langerin is problematic. It is the result and the evidence that Langerin is not a good C-type lectin receptor for the S protein. Moreover, this is in agreement with the trans-infection experiment where langerin is not efficient, as opposed to DC-SIGN and L-SIGN. As stated in the article, and contrary to many other authors studying CLRs/pathogen interaction, we did not use CLR-FC fusion proteins, that all present Carbohydrate Recognition Domain of the corresponding lectins in the same way. Here, to analyze the interaction we used the full extracellular domain of the considered lectins, with their relevant oligomeric state and thus with proper topological presentation of their CRDs. The known difference in flexibility between DC/L-SIGN CRDs and langerin CRD and the fact that DC/L-SIGN is tetrameric while langerin is trimeric (and thus with a lower potential of avidity), could fully explain the observed difference between these lectin receptors. Such significant difference would not have been seen using CLR-Fc fusion.
We already emphasized in the article the significance of using the full ECD versions of the lectin receptors. We do not think that more details are required in the text. Please see at the bottom of page 26, in the discussion, the following sentence:

> *Similarly, we expressed the entire ectodomain for the CLRs as well, avoiding Fc-CRD constructs, in order to preserve their specific oligomeric assembly and therefore their avidity properties.*

Controls to with soluble ACE2 to validate the S ectodomain protein, as well as deglycosylated S to confirm that interaction with the C-type lectin receptors is specifically

glycan dependent (may find that Langerin binding is non-specific, explaining the lack of saturation).

The interaction is glycan-dependent. The SPR surfaces are completely regenerated after EDTA treatment and regain their interaction capacity after reconstitution by a buffer containing Ca^{2+}. This is verified over a very large number of regeneration/reconstitution cycles and shows that the spike/CLRs interaction is strictly dependent on the presence of calcium in the carbohydrate binding domain. This is a characteristic of the glycan-dependent interaction with CLRs.
This dependance of binding on Ca^{2+} is also strictly observed for all the other CLRs studied. Please see below 3 of the SPR experiments for DC-SIGN, L-SIGN and Langerin but with the regeneration phase visible.
You can notice the immediate and complete regeneration of the surface after the EDTA treatment. It would not behave this way in case of non-specific protein-protein interaction.

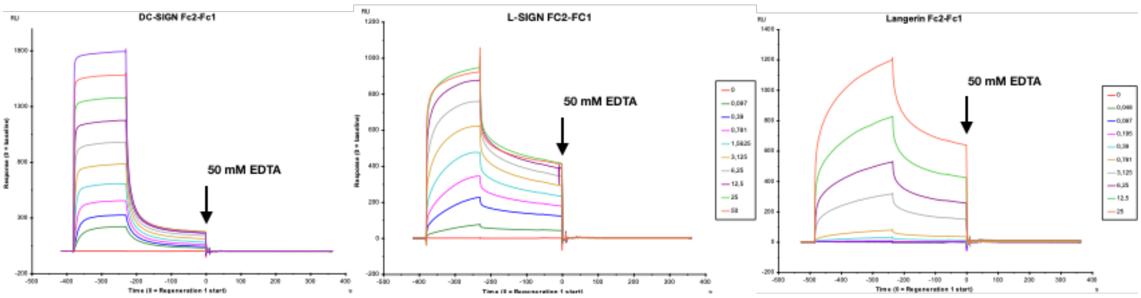

In addition, comparison of SARS-CoV-2 and SARS-CoV ectodomain would enhance the study, or as an alternative, comparison to EBOV GP ectodomain could be done and correlated to the trans-infection data.

We think it is not justified for this work. Development and production of both S protein from SARS-CoV and GP from EBOV would represent an important work and delay for this article without improving strongly the level of information. There is already some infection experiment showing that it is working with EBOV and similar behavior/properties with SARS-CoV have been observed previously (Marzi, A. et al. JVI **2004**, 78, 12090–12095).

Another issue is that the outcome of C-type lectin receptor-S binding is unknown as well as the mechanism by which trans-infection occurs. For instance, once there is C-type lectin engagement, what happens to the virions? Are they internalized? Does this lead to some degradation/inactivation of a fraction of the viral population? Are the C-type lectin receptors activated? Is trans-infection more efficient than cell-free infection? Is this unique to SARS-CoV-2 when compared to SARS-CoV?

All these questions represent completely new studies, more centered on the mechanism of trans infection itself, while in this work we described this new mode of transmission and the role of CLRs in it. It is not the scope of this study in particular. These questions are under investigation, and still now under debate, in the case of cis-and trans-infection of HIV, despite years of work. This illustrates how large are the efforts to be conducted in the future to address these points.

Part III – Minor Issues: Editorial and Data Presentation Modifications
Please use this section for editorial suggestions as well as relatively minor modifications of existing data that would enhance clarity.

Reviewer #1: I would perhaps discuss a bit more the possible location of the virus in the dendritic cell prior to trans infection. This is mentioned briefly but could help the reader understand the process a bit more. For HIV there is a debate about whether the virus simply sticks to the surface of the cell, or is actively pinocytosed into a non-endosomal compartment awaiting trans-infective release.

We thank reviewer one for this nice suggestion. We added at page 29 (bottom) the following sentence.
"Future studies will address the trans infection mechanism, whether the virus are simply attached and stabilized at cell surfaces by CLRs or internalized in dedicated compartments, awaiting trans infective release."

Reviewer #2: In the text, the authors state that they could not calculate kinetic association and dissociation rate constants (kon and koff). An inverted experiment, i.e. attach the lectin receptors to the surface and inject the spike protein, could help solve the problem

The "inverted experiment" will not help to solve the problem of access to kinetic constants, since, also in that set up, there will be multivalent interactions and thus an avidity phenomenon. However, the set up suggested by the referee will allow higher avidity, since Spike will be able to interact with more than 4 sites on the surface. Indeed, while DC-SIGN tetramer, when injected on Spike surfaces (as in fig 2), can establish at maximum 4 interactions, the spike proteins (due to their numerous N-Glycans) will allow a much higher number of interactions onto a DC-SIGN surface presenting numerous CRDs. Thus, doing so, Kd_{app} in the nanoM range instead of the µM range are expected. This set up of interaction was not possible here for technical reasons. We could attach Lectins, in an oriented way, on surface thanks to biotin/streptavidin capture. However, the Spike protein used possess 2 streptagII that would interfere also with the surface. A new Spike version without strep Tag will be developed in the future to allow such inverted SPR interaction studies. However, at this stage, this will not change the demonstration of interaction of Spike protein with C-type lectins, as presented here.

"Our work shows that DC/L-SIGN are important enhancers of infection mediated by the S protein of SARS-CoV-2 that greatly facilitate viral transmission to susceptible cells." The term "enhancers" is not the appropriated term here. Authors are not showing a "basal" infection and then the enhancement of it by the presence of DC/L-SIGN. They show trans-infection, but not an enhancement of the infection.

Thanks for this remark. We have changed the sentence in the following way:

> *"Our work shows that DC/L-SIGN are important factors contributing to additional routes of infection also mediated by the S protein of SARS-CoV-2. This trans infection process greatly facilitates viral transmission to susceptible cells."*

Histograms with SEM of all figures should be represented as scatter plots with each point representing one individual replicate for better appreciation of variations.

Figures have been modified to show scatter plots of individual results

The authors conclude "the specific topological presentation of their CRD as well as their oligomeric status is preserved for each of the CLR". This conclusion is based on

the use of recombinant constructs corresponding to the extracellular domains. Additional experiments are needed to verify if these properties are retained in the entire protein or reference that support this conclusion is needed. If not, this sentence should be rephrased.

The oligomeric and/or structural characterization of those ECD domain have been detailed previously in other studies available. We added the corresponding reference at the end of this sentence.

Fig. 1: Transmission Electron Microscopy images in G. Is there some protein aggregation at this time point ?

There is no visible protein aggregation observed at the time point G.

If so, could the authors confirm that for these studies, proteins at day 22 were not used?

No, we used freshly made preparation of Spike for the SPR
For EM, final size exclusion chromatography and EM grid were done in sequence.

In a supplementary figure, the authors should show what is described in the text, “twenty out of the twenty-two SARS-CoV-2 S protein N-linked glycosylation sequons” conserved, when compared to SARS-CoV-1 S.

Fig2:
- Negative controls should be shown as supplementary information. Ex: Lectins alone, do they attach to the surface?

All sensorgrams presented are subtracted from the surface controls. (see figure below). Here Fc1 is functionalized with a non-glycosylated protein, BSA, while the other surface is functionalized with the spike protein. Upon injection onto those surfaces that are in series, the same lectin solution is flowing on the control surface (Fc1, sensorgram on the left below) and the sample surface, with spike (Fc2 here, sensorgram in the middle). The signal that is considered for interaction is the difference resulting from the subtraction of Signal on Fc1, corresponding to bulk effect (clearly, the case here) and/or potential non-specific interaction with the surface (not the case here), to the signal of interaction with the spike from Fc2 (see the resulting sensorgram Fc2-Fc1, below on the right)
This is really routine treatment in SPR, we do not think that it is justified here to add such detailed sensorgrams for all the SPR experiments in the supp info. One example is added here for your evaluation. You can see that there is no significant interaction on the control surface (on the left)

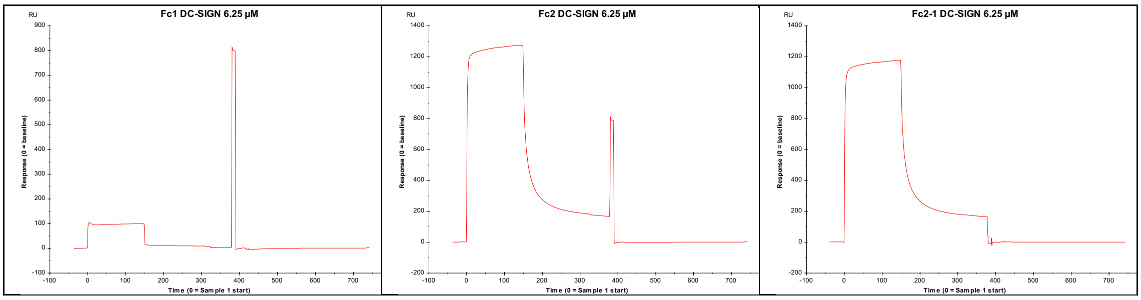

In the, “spr binding studies” section of the “materials and methods”, we added a precision (into brackets and italic here) to the end following sentence:
“The resulting sensorgrams were reference surface corrected *(subtractions from Fc1 signal)*”.

- The interaction of Spike and DC/L-SIGN is indeed verified here in vitro, which is the main point of the figure. If possible this result should be confirmed using pseudotyped viruses.

Not possible to do it : not allowed to inject virus in the SPR machine of the platform of our institute. Moreover, it would require highly pure virion preparations to avoid of any pieces of cellular membrane that may also harbor glycosylation or glycoconjugates. Very complicated experiment to do, if you want it clean.

Fig3:
- In A and B, the models of the two proteins observed by TEM are represented. It would be interesting to also show a model of the complex that is observed by TEM in C.

The choice of a specific representation of one such complex among the many possibilities may not be easily to justified in the main text. However, it can be probably more acceptable in the graphical abstract of the article, where more liberty is possible. Thus, such a DC-SIGN/Spike complex will be presented as a graphical abstract. We thank the referee for this suggestion.

- In the text the authors say they are expecting a stoichiometric Spike/CLR complex of 1:1, but for the TEM experiments they use a ratio of 1:3 (1 trimeric spike for 3 tetrameric DC-SIGN ECD). Is there a specific reason for this? If so, this should be commented in the discussion. This information might be useful for other people who would want to do similar experiments.

To enrich in complex, we use an excess of CLRs, before EM we did a SEC to purify the complex and eliminate the excess of DC-SIGN. An additional figure about this experiment is added in the supp info see below.

- SEC profiles should be shown as supplemental figures. It is valuable information for other researchers who might want to do experiments related to these results.

SEC profiles have been added as asked by the referee as supplemental figures

Fig4:
- The results presented in this figure should be repeated in a more relevant model: Calu-3, A549 expressing ACE2, or other respiratory cell lines expressing ACE2.

The respiratory Calu-3 cell line has now been used in combination with authentic SARS-CoV-2 (see above and Figure 7)

- In panel A, authors used MDDC and M2-MDC cells, while in panel B, the trans-infection is shown only in MDDC cells. Were experiments in M2-MDC done as well? If so, results should be added here. In particular, as in the discussion, authors conclude on both cell lines. If there are results on M2-MDC, they should appear in Fig. 4, along with MDDC trans-infection. If not, the conclusion (lines 533-536) needs to be restricted to MDDCs.

That’s correct, no M2 Macrophages were used in Fig4B due to the lack of cells, the conclusion line (now 605) has been corrected as requested

- Data showing DC/L-SIGN expression in these cells should be provided.

Expression of DC-SIGN by derived cells has been extensively proved and the protocol is a standard followed by many laboratories. Nevertheless, DC-SIGN expression on MDDC by using a specific labeled antibody in cytometric analysis is now shown as Supplementary Figure 3.

- More details should be provided in the legend to understand the figure.

The legend has been revised for clarity

Fig5:

- More details should be provided in the legend to understand the figure.

The legend has also been revised for clarity

- Line 377: “Interestingly, no trans-infection was observed using Jurkat Langerin cells.” Why is it interesting? This was expected from the SRP results.
- Sentence in the discussion “On the other hand, DC/L-SIGN expression on Jurkat cells allows binding and capture of SARS-CoV-2 pseudovirions”. There is no experiment showing the binding and capture of pseudovirions in these cells.

That’s right related to binding. We, also eliminated the term “capture” from the sentence.

Fig6:

- Another major comment: this is one of the most important figures of the paper, or at least it is important for the conclusion about using PM26 as an inhibitor to decrease the spread of the infection. However, the experiments related to the inhibition (panels C and D) seem to have been performed only once. These results must be replicated in at least 3 independent experiments.

A third independent experiment for panel C is added and new competition experiments using PM26 have been added in the article BUT in the new section using this time against authentic SARS-CoV-2 virus and using Calu3 as a cell target for infection.

For panel C, a third independent experiment of PM26 inhibition of the interaction between DC-SIGN/Spike has been added.

- Authors use PM26 as an antagonist for DC-SIGN. Is there a similar mannoside for L-SIGN. If so, it would be relevant to perform similar experiment with it. In particular, as L-SIGN seems to show a better trans-infection rate than DC-SIGN (Fig 5C).

No, this glycomimetic has been developed with a specificity towards DC-SIGN. No such compounds are available up to now for L-SIGN; Maybe something new to develop for the future.

- It is written that the oriented and non-oriented Spike set-ups were used for the SPR competition assays, but in panel B, there is only the oriented one. sensorgrams of the interaction with the non-oriented set-up should be added.

We have added the required sensorgrams in supp info.

- Some details are missing to entirely understand the figure without extensively referring to the material and methods or results sections.

We have performed a new set of experiments that are now replacing panel D. The legend has been modified accordingly.

In the discussion, it is said that type II alveolar cells are known to express high levels of L-SIGN. If type II alveolar cells, believed to express ACE2, express also L-SIGN, would there be a trans-infection phenomenon here, or L-SIGN could help to attract and bind Spike, so then ACE2 could properly bind the latter? Maybe something to add to the discussion.

A comment on the effect of the combination of the lectin and the ACE2 receptor on the same cell has been added in the discussion: currently unknow and worth exploring.

Lines 565-567: “DC/L-SIGN expression can be upregulated as well, since it has been demonstrated that while innate immune responses are potently activated by SARS CoV-2, it also counteracts the production of type I and type III interferon”. The sentence is confusing. Has it been shown that DC/L-SIGN is upregulated by SARS-CoV-2 infection or is it an assumption from the fact that IFN-I and III are downregulated during a SARS-CoV-2 infection?

Yes, the sentence refers to a potential upregulation of DC/L-SIGN in the context of low levels of IFN by SARS-CoV-2 infection

Lines 584-587: “Additionally, upon binding to DC-SIGN PM26 was shown to induce a pro-inflammatory anti-viral response, which should be beneficial at the onset of the infection and may help to steer the immune response towards a profile correlated 586 with milder forms of the disease.” Either there is something wrong with the sentence or a deeper discussion of this idea needs to be provided. As mentioned by the authors, the cytokine storm is associated with SARS-CoV-2 pathogenesis. It is therefore difficult to understand why one would use a pro-inflammatory molecule in patients that would already have an initiated proinflammatory response?

The exact timing on the establishment of an effective response is thought to be an important issue here. Activation of innate immunity at the onset of the infection process could be beneficial to control further replication and dissemination of SARS-CoV-2. On the contrary, proinflammatory activation would no longer be desirable once severe disease is established.

Final paragraph of the discussion “Demonstration of involvement of APCs in SARS-CoV-2 early dissemination through CRLs DC/L-SIGN…”. This is clearly an overstatement. There is no demonstration of this in this study. The presented results may at most suggest a model. As discussed above further, more physiologically relevant experiments are required to draw such statements.

We accept that this is a model of involvement of relevant APCs on COVID-19 pathogenesis based in CLRs expression and now the statement has been modified accordingly.

The last sentence of the abstract should be modified: the authors should use respiratory cell lines and not Vero E6 cells to conclude on a potential spreading of infection.

We do agree with referee, but since we have now used Calu3 cells and got same behavior/data, we can finally keep the statement.

Minor comments related to the format:
There are some sentences/ideas where references are missing:
Lines 67-75,
done
line 107 (transmission rates),
done
lines 110-117,
done
lines 431-437,
done
lines 446-448,
done
lines 455-457,
done
lines 463-465,
done
lines 498-499,
done
lines 515 (HIV).
done
Unformatted reference at line 573-574.
Pb corrected

Line 562: replace though by through?
Corrected, thanks

Line 581: replace Vero cells by Vero E6 cells.
Modified

Use C-type lectin receptors (CLR) abbreviation throughout the text after first definition.
Done

Definition of DC or L-SIGN abbreviation was not introduced.
Done on their first appearance in the introduction

Sentence at lines 99-101 reads oddly. Do the authors mean that the cleavage at S2' site is necessary to trigger conformational changes in S2? If so, I think it is difficult to understand that idea with the sentence as it is written.
It has been modified.

Sentence at line 493: “DC-SIGN can also bind to some o complex N-glycosylation sites”. What does it mean some "o" complex?

It should have been “other”. It has been corrected. Thank you

Reviewer #3: In the introduction, the authors hypothesized that difference in binding of attachment factors (such as C-type lectin receptors), may play a role in differential transmission rate of SARS-CoV-2 when compared to SARS-CoV (lines 107-110). This led me to think that this study will provide sought-after comparative data of SARS-CoV and SARS-CoV-2, yet only SARS-CoV-2 is investigated here, and this study does test the hypothesis. As mentioned above, data with SARS CoV ectodomain would greatly enhance this study. The mention of the authors’ hypothesis puts the spotlight on the absence of data with SARS-CoV.

Data with SARS-CoV are already accessible since the SARS outbreak from 2003 (see ref 13 to 15 of the manuscript). Moreover, we do not have yet such expression construct for S protein from SARS-CoV. So even if, as It would represent significant amount of work without bringing new significant data.

In addition, although I agree with the authors that the use of the full ectodomain is more likely to give biologically relevant data than the RBD alone (lines 462-463), it is important to note that an uncleaved and stabilized version was used and some caution should be taken when interpreting the data. Was a stabilized version of the spike used for the VSV pseudotypes? This is not mentioned in the materials and methods.

The wild type spike protein was used for pseudovirus construction. A stabilized version of spike wouldn’t be infective

In line 137, virulence is inaccurate, at least for the D614G variant (better at replicating and infecting upper airway epithelial cells, potentially better transmitted, but no clear effect on virulence). Please provide references for an effect on virulence.

That’s correct. Transmission is the right term and has been corrected.

Please, provide p values for the infection data (Figs.4-6).

Statistical significance has been calculated and p values are shown in Figures 4-7

Some sections of the discussion are too speculative and are not based on the data presented (Lines 561-576).

we think that COVID-19 pathogenesis and its association with immune response and inflammation are obviously not completely clear
and subjected to discussion, nevertheless, these lines have been now edited for clarity and concision:

Line 78, add “enveloped” in front of viruses. This statement only applies to them.
done
